# Arbeitsunfähigkeit bei psychischen Störungen – ökonomische, individuelle und behandlungsspezifische Aspekte

**DOI:** 10.1007/s00103-024-03894-6

**Published:** 2024-05-24

**Authors:** Lena Melzner, Christoph Kröger

**Affiliations:** Institut für Psychologie, Abteilung für Klinische Psychologie und Psychotherapie, Universität Hildesheim, Universitätsplatz 1, 31141 Hildesheim, Deutschland

**Keywords:** Gesundheitsökonomie, AU-Tage, Selektionshypothese, Kausationshypothese, Arbeitsplatzbezogene Psychotherapie, Health economics, Sick-leave days, Selection hypothesis, Causation hypothesis, Work-related treatment

## Abstract

Mit den Veränderungen der modernen Arbeitswelt gehen Belastungen einher, die die psychische Gesundheit von Arbeitnehmenden negativ beeinflussen können. Im Einklang damit zeigt sich ein Anstieg des Anteils der Arbeitsunfähigkeits(AU)-Tage aufgrund von psychischen Störungen auf zuletzt 17,7 % im Vergleich zu 10,9 % im Jahr 2007, womit 2021 Kosten in Höhe von 42,9 Mrd. € aufgrund von Bruttowertschöpfungsverlusten und Produktionsausfällen verbunden waren.

Dieser Artikel gibt anhand aktueller gesundheitsökonomischer Studien einen Überblick über die volkswirtschaftlichen Auswirkungen von Arbeits- und Erwerbsunfähigkeit aufgrund psychischer Störungen in Deutschland. So sind in absoluten Zahlen die Ausgaben für Arbeitsunfähigkeit insbesondere bei häufigen psychischen Erkrankungen, wie affektiven und Angststörungen, hoch. Seltenere psychische Störungen, wie die posttraumatische Belastungsstörung (PTBS) und Essstörungen, verursachen im Verhältnis zu ihrer geringen Prävalenz insbesondere hohe Kosten aufgrund von Krankengeldzahlungen.

Neben diesen wirtschaftlichen Implikationen werden die Konsequenzen von Arbeits- und Erwerbsunfähigkeit sowie Arbeitslosigkeit auf individueller Ebene beleuchtet und Erklärungsansätze vorgestellt. Letztere verdeutlichen die Notwendigkeit wirksamer Behandlungsmethoden. Dabei haben sich anerkannte Therapieverfahren als effizient in der Reduktion von AU-Tagen erwiesen. Dies gilt umso mehr für arbeitsplatzbezogene Interventionen, die dahin gehend konventionellen Verfahren überlegen zu sein scheinen. Arbeitsplatzbezogene Therapieverfahren legen einen Fokus auf die Planung der Wiedereingliederung. Weitere naturalistische Studien sind nötig, um die Übertragbarkeit der Wirksamkeit der Behandlungsmodelle auf andere Störungsbilder überprüfen zu können.

## Abstract (en)

The changes in the modern work environment are accompanied by specific stressors that can have a negative impact on employees’ mental health. In line with this, the proportion of sick-leave days due to mental disorders has recently risen to 17.7% compared to 10.9% in 2007, which in 2021 was associated with costs of 42.9 billion euros due to losses of gross value and productivity.

Based on current health economic studies, this review provides an overview of the economic impact of incapacity to work and early retirement due to various mental disorders in Germany. In absolute figures, expenditure on incapacity to work is particularly high for common mental illnesses such as affective and anxiety disorders. Rarer mental disorders such as post-traumatic stress disorder and eating disorders cause high costs in relation to their low prevalence, particularly due to sickness benefit payments.

In addition to these economic implications, the consequences of incapacity to work, early retirement, and unemployment are examined at an individual level and explanatory approaches for the specific psychosocial stresses are presented. The latter highlights the need for scientifically substantiated treatment methods. Certified treatments have proven to be efficient in reducing the number of sick-leave days, particularly for common mental disorders. This applies even more to workplace-related interventions, which appear to be superior to conventional methods in this respect. Workplace-based therapies incorporate work-related models and focus on the planning of reintegration into the workplace. Further naturalistic studies are needed to test the transferability of the effectiveness of these treatments to other disorders.

## Hintergrund: Stellenwert der Arbeit und Wandel der Arbeitswelt

Schon allein zeitlich nimmt die Arbeit eine herausgehobene Stellung in unserem Leben ein. So werden Menschen, die im Jahr 2021 15 Jahre alt waren, statistisch gesehen ca. 39 Jahre arbeiten [1], was bei einer mittleren Lebenserwartung von 81 Jahren [2] etwa 62 % des Erwachsenenlebens entspricht. Dabei betrug die wöchentliche Arbeitszeit 2022 im Mittel 34,7 h (29,5 % in Teilzeit: 20,8 h; Vollzeit: 40,4 h; [3]). Bei einer 5‑Tage-Woche verbringen Arbeitnehmende in Vollzeit also ca. ein Drittel des Tages mit der Erledigung von beruflichen Tätigkeiten. Im Einklang damit rangiert in einer repräsentativen Befragung der Konrad-Adenauer-Stiftung [4] die Kategorie „Beruf und Arbeit“ unter den als sehr wichtig eingeschätzten Lebensbereichen mit 42 % auf einem ähnlichen Niveau wie „Freunde und Bekannte“ (45 %) sowie „Freizeit und Erholung“ (46 %).

Arbeit erfüllt dabei neben der reinen Sicherung des Einkommens unterschiedliche psychosoziale Funktionen: Neben der Zeitstrukturierung ermöglicht der Arbeitsplatz über den Kontakt zu anderen Menschen den Ausbau kooperativer Fähigkeiten. Durch diese Kooperation kann ein Gefühl sozialer Anerkennung entstehen. Darüber hinaus dient die Arbeit dem Erwerb von Fähigkeiten und Fertigkeiten und kann so ein Kompetenzerleben schaffen, was das Selbstwirksamkeitserleben stärkt. Außerdem tragen die Erfüllung der Arbeitsaufgaben und die Einnahme einer beruflichen Rolle zur Herausbildung der persönlichen Identität und zur Steigerung des Selbstwertgefühls bei [5].

Nichtsdestoweniger gehen die zunehmenden Veränderungen der Arbeitswelt auch mit spezifischen Belastungen von Arbeitnehmenden einher. Auch wenn die Entwicklung in den letzten Jahren stagnierte, nahmen in der Vergangenheit die atypischen, sog. prekären Arbeitsverhältnisse wie Zeitarbeit, befristete Arbeitsverhältnisse und Minijobs [6] sukzessive zu, was eine sog. Fragmentierung der Erwerbsbiografie [7] mit Berufswechseln und genereller Arbeitsplatzunsicherheit bedingt. Daneben verstärkt die Tertiarisierung, also die Zunahme von Beschäftigten im Dienstleistungssektor, die psychologischen Anforderungen an Beschäftigte, u. a. durch die Flexibilisierung der Arbeit und Arbeitsverdichtung [8]. Im Rahmen der Globalisierung steigt außerdem der Wettbewerbsdruck, womit u. U. erhöhte Mobilitätsanforderungen, die Anforderung ständiger Erreichbarkeit und eine wachsende leistungsabhängige Gehaltsdifferenzierung verbunden sind.

Diese Transformationen in der modernen Arbeitswelt können ein erhöhtes Stresserleben bedingen und sich negativ auf die psychische Gesundheit der Arbeitnehmenden auswirken, was die Entwicklung psychischer Störungen begünstigt (z. B. [9, 10]). Damit einher geht ein steigender Anteil an Arbeitsunfähigkeitstagen^1^ (AU-Tagen) aufgrund psychischer Störungen. So gingen im Jahr 2021 17,7 % der bei den Krankenkassen gemeldeten AU-Tage auf psychische und Verhaltensstörungen zurück^2^ ([11]; Versicherte der Allgemeinen Ortskrankenkassen, Betriebskrankenkassen und Ersatzkassen), der zweithöchste Anteil nach Erkrankungen des Muskel-Skelett-Systems und des Bindegewebes, wodurch hohe gesundheitsökonomische Kosten entstehen.

Im vorliegenden Artikel soll neben der damit einhergehenden volkswirtschaftlichen Relevanz der Arbeits(un)fähigkeit ein weiterer Fokus auf deren individuelle Auswirkungen für die betroffenen Personen gelegt werden. Darüber hinaus erfolgt ein erster Überblick über Behandlungsprogramme, welche die (Wiederherstellung der) Arbeitsfähigkeit einbeziehen.

## Gesundheitsökonomische Aspekte von Arbeitsunfähigkeit und Erwerbsminderung

**Abb. 1:**
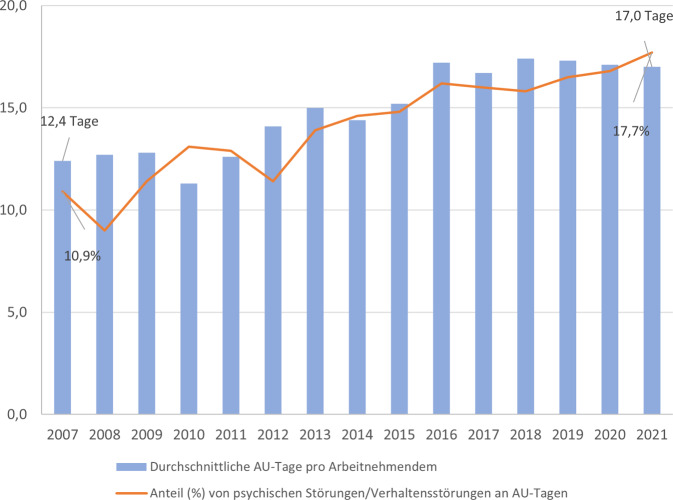
Durchschnittliche jährliche Arbeitsunfähigkeitstage (*AU-Tage*) pro Arbeitnehmendem (*blaue Balken*) und Anteil psychischer Störungen und Verhaltensstörungen an diesen AU-Tagen (*orange Linie*) von 2007 bis 2021 (*Quelle: *eigene Darstellung auf Basis der jährlich erscheinenden Berichte „Sicherheit und Gesundheit bei der Arbeit“ (*SuGA*) der Bundesanstalt für Arbeitsschutz und Arbeitsmedizin; [13])

**Abb. 2:**
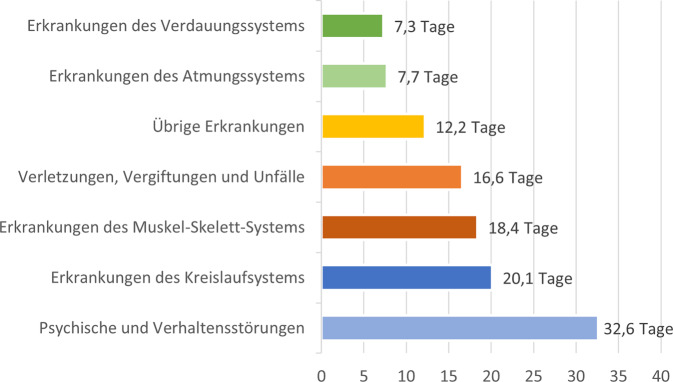
Durchschnittliche Arbeitsunfähigkeitstage (*AU-Tage*) je Diagnose nach Diagnosegruppen (*Quelle:* eigene Darstellung nach dem Bericht „Sicherheit und Gesundheit bei der Arbeit 2021“ (*SuGA*) der Bundesanstalt für Arbeitsschutz und Arbeitsmedizin; [11])

Seit Anfang/Mitte der 2010er-Jahre zeigt sich je nach Quelle ein leichter bis moderater Anstieg in der Anzahl der AU-Tage insgesamt ([12, 13]; Abb. 1), der in den letzten Jahren jedoch stagnierte.^3^ Während die AU-Tage bei den Versicherten der Allgemeinen Ortskrankenkassen, Ersatzkassen und Betriebskrankenkassen von 2012 bis 2021 insgesamt um 20,6 % zunahmen, ist der Anteil psychischer und Verhaltensstörungen an diesen im selben Zeitraum relativ stetig um 55,3 % angestiegen [13]. Dies ist insbesondere auf die zunehmende Dauer der Krankschreibungen aufgrund psychischer Störungen zurückzuführen. So war die Dauer der Arbeitsunfähigkeit pro Diagnose in dieser Erkrankungsgruppe im Jahr 2021 mit Abstand am längsten (*M* = 32,6 Tage; Abb. 2) und fast doppelt so hoch wie bei der häufigsten Erkrankungsgruppe (*M* _*Muskel-*_ _*und Skeletterkrankungen*_ = 18,4 Tage). Außerdem sind psychische Störungen nach Muskel- und Skeletterkrankungen der zweithäufigste Grund für Langzeit-Arbeitsunfähigkeiten [14], welche durch den Bezug von Krankengeld zusätzlich hohe indirekte Kosten verursachen^4^ (Tab. 1), und sie stellen die häufigste Ursache für Frühverrentungen aufgrund von Erwerbsunfähigkeit dar.

**Tab. 1:** Schätzungen jährlicher gesundheitsökonomischer Kosten durch verbreitete psychische Störungen in Deutschland

Studie	Zeitraum Datensammlung	Datenquellen	Störungsbilder	Prävalenz (%)	AU-Tage/Person	Kosten durch AU in Euro (Verluste/Ausfälle)	Anzahl Frühverrentungen	Kosten durch Frühverrentungen in Euro	Krankengeldtage/Person	Kosten durch Krankengeld in Euro
Wunsch et al., [17]	2010	DRV; InEK- Datenbanken; AOK BKK, DAK und TK (Gesundheitsberichte); Statistisches Bundesamt	Affektive Störungen	12,9 [61]	6,1	Produktion: 2,2 Mrd.; Bruttowertschöpfung: 5,6 Mrd. *Gesamt: 7,8* *Mrd.*	11.766	273,7 Mio.	2,6	568,1 Mio.
			Angststörungen	14,5 [61]	4,9	Produktion: 2,1 Mrd.; Bruttowertschöpfung: 5,6 Mrd. *Gesamt: 7,7* *Mrd.*	5766	134,1 Mio.	2,1	559,1 Mio.
Wunsch et al., [20]	2012	DRV; AOK, BKK, DAK und TK (Gesundheits-berichte); GBE-Bund; BMG-Veröffentlichungen; VGRdL; Bundesagentur für Arbeit	Borderline-Persönlichkeitsstörung	0,7 [62]	13,4^*a*^	Produktion: 179,7 Mio.; Bruttowertschöpfung: 314,1 Mio. *Gesamt: 493,8* *Mrd.*	1197	27,8 Mio.	8,9	54,8 Mio.
Bode et al., [19]	2010–2014	DEGS1-MH; Statistisches Bundesamt; BAuA; Bundesfinanzministerium; BfR	Anorexia nervosa	0,7 [21]	26,4	Produktion: 749,2 Mio.; Bruttowertschöpfung: 1,3 Mrd. *Gesamt: 2,1* *Mrd.*	314	7,3 Mio.	20,7	308,1 Mio.
			Bulimia nervosa	0,2 [21]	24,2	Produktion: 196,5 Mio.; Bruttowertschöpfung: 343,3 Mio. *Gesamt: 539,8* *Mio.*	24	558.242	17,8	76,2 Mio.

Allen Kosten dient die erwerbstätige Bevölkerung als Berechnungsgrundlage, mit Ausnahme der Krankenhausaufenthalte (gesamte erwachsene Bevölkerung). Da teilweise nur die möglichen Kostenersparnisse durch die Behandlung der Störungen explizit angegeben werden, handelt es sich an diesen Stellen um eigene Berechnungen der Kosten anhand der in den Studien veröffentlichten gesundheitsökonomischen Daten.

*AOK* Allgemeine Ortskrankenkassen, *AU* Arbeitsunfähigkeit, *BAuA* Bundesanstalt für Arbeitsschutz und Arbeitsmedizin, *BfR* Bundesinstitut für Risikobewertung, *BKK* Betriebskrankenkassen, *BMG* Bundesministerium für Gesundheit, *DAK* Deutsche Angestellten-Krankenkasse, *DEGS1-MH* Studie zur Gesundheit Erwachsener in Deutschland, Zusatzmodul „Psychische Gesundheit“, *DRV* Deutsche Rentenversicherung, *GBE-Bund* Gesundheitsberichterstattung des Bundes, *InEK* Institut für das Entgeltsystem im Krankenhaus, *TK* Techniker Krankenkasse, *VGRdL* Volkswirtschaftliche Gesamtrechnungen der Länder

^a^Schätzungen indirekter Kosten beziehen sich hier nur auf die 35 % der Personen mit Borderline-Persönlichkeitsstörung, die berufstätig sind.

Schätzungen der volkswirtschaftlichen Kosten von Arbeitsunfähigkeit aufgrund psychischer Störungen belaufen sich auf zuletzt 15,8 Mrd. € aufgrund der reinen Lohnkosten (Produktionsausfälle) sowie 27,1 Mrd. € aufgrund des Verlusts an Arbeitsproduktivität (Bruttowertschöpfungsausfälle) jährlich [11], was im Jahr 2021 1,1 % des Bruttonationaleinkommens entsprach. Hinzu kommen Produktivitätseinbußen bei psychisch erkrankten Arbeitnehmenden, die trotz der Einschränkung am Arbeitsplatz erscheinen (sog. *Präsentismus*). Hierdurch entstehende ökonomische Belastungen sind schwer zu beziffern, liegen aber nach Schätzungen noch um ein Vielfaches höher als die Kosten aufgrund des Fernbleibens vom Arbeitsplatz (z. B. [16]).

In den letzten 10 Jahren sind einige gesundheitsökonomische Studien entstanden, die indirekte und teilweise auch direkte Kosten psychischer Störungsbilder in Deutschland adressieren und teils Kosten-Nutzen-Analysen (unterschiedlicher) psychotherapeutischer Behandlungen mit einbeziehen. Diese Erhebungen erlauben über die Daten der großen deutschen Krankenversicherungen, der Deutschen Rentenversicherung und anderer Institutionen die Betrachtung eines differenzierteren jährlichen Kostenprofils. Sie wurden über eine Recherche in den psychologischen Datenbanken PsycInfo, PSYNDEX, PubPsych und PsyJournals identifiziert; Studien mit prävalenzbasiertem Ansatz sind in Tab. 1 zusammengefasst. Die Ergebnisse zeigen, dass häufige psychische Störungen wie Angst- und affektive Störungen in absoluten Zahlen jeweils Kosten in hoher einstelliger Milliardenhöhe durch Arbeitsunfähigkeit verursachen [17], zumal die 12-Monats-Prävalenzen über 10 % betragen [18]. Jedoch weisen seltenere psychische Erkrankungen wie Essstörungen (Anorexia/Bulimia nervosa, AN/BN; [19]) sowie die Borderline-Persönlichkeitsstörung (BPS; [20]) mit Prävalenzraten unter 1 % eine vielfach höhere Anzahl an AU-Tagen pro betroffener Person auf (*M*_*Angst*_ = 4,9, *M*_*affektiv*_ = 6,1 vs. *M*_BPS_ = 13,4; *M*_AN_ = 26,4, *M*_BN_ = 24,2), sodass sie verhältnismäßig ebenfalls hohe volkswirtschaftliche Belastungen darstellen. Dies zeigt sich insbesondere für die AN, die trotz der niedrigen angenommenen Prävalenz von 0,7 % [21] mit 2,1 Mrd. € ca. ein Viertel der jährlichen Verluste durch Arbeitsunfähigkeit aufgrund von affektiven bzw. Angststörungen verursacht [19]. Diese Befunde stehen im Einklang mit früheren Forschungsergebnissen, welche für Essstörungen hohe Raten an Absentismus feststellten [22] und die BPS insgesamt als eine der teuersten psychischen Störungen charakterisieren [23]. Die in Tab. 1 dargestellten Daten verdeutlichen auch die lange Dauer von Arbeitsunfähigkeiten bei diesen Störungsbildern; die Krankengeldtage liegen bei der AN etwa 9‑mal so hoch wie bei affektiven Störungen, bei der BPS betragen sie nach den vorliegenden Daten etwa das 3,5Fache. Entsprechend zeigen sich auch relativ hohe Raten an Frühverrentungen bei betroffenen Personen, zumal diese bei längerer Dauer der Arbeitsunfähigkeit und mit zunehmendem Alter wahrscheinlicher werden.

Absolut sind die Kosten von Krankengeldbezügen und Frühverrentungen jedoch insbesondere bei affektiven und Angststörungen volkswirtschaftlich relevant. So betrugen die Krankengeldzahlungen bei beiden Störungsgruppen jeweils ca. 550 Mio. € jährlich, bei den Frühverrentungen zeigten sich mit 273 Mio. € bei affektiven Störungen mehr als doppelt so hohe Kosten wie bei Angststörungen. Anzumerken ist insgesamt, dass die Kosten der einzelnen Störungsbilder angesichts des allgemeinen Anstiegs an AU-Tagen und Krankengeldtagen aufgrund psychischer Störungen seit Ende der Erfassung im Rahmen der aufgeführten Studien noch einmal angestiegen sein könnten, obwohl sich die 12-Monats-Prävalenz psychischer Störungen in Deutschland in den letzten Jahrzehnten relativ stabil im Bereich von ca. 25–33 % bewegt [18, 24, 25].

Neben diesen prävalenzbasierten Ansätzen wurden in jüngerer Vergangenheit zwei deutsche Studien zu Essstörungen bzw. posttraumatischer Belastungsstörung (PTBS) mit inzidenzbasierten Ansätzen veröffentlicht [26, 27], in denen retrospektiv die – direkten und indirekten – Kosten im Zeitraum um das Jahr der Indexdiagnose betrachtet wurden. Sie erlauben die Identifikation von Trends in der Kostenentwicklung. Basierend auf Daten der gesetzlichen Krankenversicherungen zeigte sich bei Versicherten mit PTBS im Vergleich zu einer gematchten Gruppe ohne diese Diagnose jeweils ein steiler Kostenanstieg über die 2 Jahre bis zur Indexdiagnose [27]. Die Kosten aufgrund von Arbeitsunfähigkeit zeigten in den 2 Jahren nach Indexdiagnose wieder einen entsprechenden Abwärtstrend, blieben jedoch über dem Ausgangsniveau. Der Vergleich mit der Nicht-PTBS-Gruppe stellt heraus, dass die Kosten für diese Gruppe auf einem ähnlichen Niveau starten, jedoch hier über die 5‑jährige Analyseperiode nur leicht ansteigen, was die hohen inkrementellen ökonomischen Belastungen durch Arbeitnehmende mit PTBS verdeutlicht. Dies stimmt mit früheren Forschungsergebnissen überein, welche die PTBS mit hohen sozialen Kosten assoziieren [28]. Eine geschlechtervergleichende Analyse der Kostenentwicklung bei AN und BN ergab einen ähnlichen Trend, wobei auffällig war, dass Personen mit BN geschlechterunabhängig vor und nach der Indexdiagnose höhere Bruttowertschöpfungsverluste aufwiesen als Personen mit AN [26].

Bezüglich spezifisch arbeitsbezogener Ängste zeigte eine aktuelle repräsentative Erhebung ebenfalls erhebliche indirekte Kosten [29]: So gingen hohe Ausprägungen selbstberichteter arbeitsphobischer Ängste im Vergleich zu kleinen Ausprägungen mit deutlich längeren Arbeitsunfähigkeitszeiten in den vergangenen 12 Monaten einher. Außerdem berichteten Personen mit hoch ausgeprägten arbeitsphobischen Ängsten die größte Anzahl an Arbeitslosigkeitsperioden über die Lebensspanne und zeigten zu 42 % eine sehr hohe Beeinträchtigung in der Widerstands- und Durchhaltefähigkeit am Arbeitsplatz. Demzufolge darf auch infolge arbeitsbezogener Ängste von erheblichen volkswirtschaftlichen Einbußen ausgegangen werden, wobei konkrete Analysen hierzu noch ausstehen.

Insgesamt verdeutlichen die dargestellten Analysen die gesundheitsökonomische Notwendigkeit, effiziente Behandlungsmodelle, ggf. mit Arbeitsplatzfokus, für Arbeitnehmende zu entwickeln und möglichst in kontrollierten Studien zu erproben.

## Individuelle Auswirkungen von Arbeitsunfähigkeit, Erwerbsminderung und Arbeitslosigkeit

Auch auf persönlicher Ebene geht Arbeitsunfähigkeit mit vielfältigen Einschränkungen für die betroffenen Personen einher. Wie bereits einleitend beschrieben, erfüllt die Arbeit neben der rein finanziellen Versorgung weitere wichtige psychische sowie auch soziale Funktionen. So können vor allem die Einschränkungen sozialer Teilhabe einen Verstärkerverlust bedingen. Zahlreiche Studien zeigen, dass sowohl Arbeitsunfähigkeit als auch insbesondere Frühverrentungen und Arbeitslosigkeit mit einer insgesamt niedrigeren Lebenszufriedenheit sowie höheren psychischen Belastungen einhergehen [30]. Betrachtet man erwerbsunfähige, frühverrentete Personen, zeigt sich darüber hinaus eine erhöhte Mortalitätsrate sowie ein Anstieg der Suizidraten [31, 32]. Langzeit-Arbeitsunfähigkeit, bei der die Krankengeldzahlungen nur 70 % (§ 47 SGB V) des vorherigen Arbeitsentgelts betragen, bringen außerdem erhebliche finanzielle Einbußen mit sich, welche die Lebensführung beeinträchtigen können. Aktuelle Daten zeigen für die Gruppe der Arbeits- und Erwerbslosen die mit Abstand höchste Armutsgefährdungsquote^5^: Mit 49,4 % ist etwa die Hälfte dieser Personen von Armut bedroht, während es bei den Erwerbstätigen nur 8,9 % sind [33].

In Selbsteinschätzungen geben Arbeitslose darüber hinaus eine schlechtere körperliche und psychische Gesundheit an und weisen auch objektiv eine höhere Wahrscheinlichkeit auf, an häufigen psychischen Störungen wie Angststörungen, affektiven Störungen sowie substanzbezogenen Störungen zu erkranken als Erwerbstätige [34, 35]. Sowohl Krankenversicherungsdaten [36] als auch Selbstauskünfte zeigen außerdem eine höhere Inanspruchnahme des Gesundheitssystems in Form von Arztbesuchen und Krankenhausaufenthalten [37, 38], wobei dieser Zusammenhang durch die schlechtere körperliche und psychische Gesundheit mediiert sein könnte [39]. Im Einklang damit zeigen sich bei Arbeitslosen ebenfalls höhere Mortalitäts- und Suizidraten als in der Allgemeinbevölkerung [40, 41]; dies gilt umso mehr, je länger die Arbeitslosigkeit andauert [42].

Die dargestellten gesundheitlichen Einbußen beziehen sich auf arbeits- und erwerbsunfähige sowie arbeitslose Personen im Allgemeinen. Auch wenn konkrete Daten für die Personengruppe fehlen, erscheint aufgrund der höheren grundlegenden Belastung von Menschen mit psychischen Störungen die Schlussfolgerung naheliegend, dass diese durch ein längeres Fernbleiben vom Arbeitsplatz u. U. noch stärker belastet sein könnten.

Es gibt unterschiedliche Erklärungsansätze dazu, warum ein längeres Fernbleiben vom Arbeitsplatz negativ mit der psychischen Gesundheit zusammenhängt. Die *Kausationshypothese* besagt dabei, dass mit dem Verlust des Arbeitsplatzes spezifische, psychosoziale Belastungen einhergehen, die Verschlechterungen der mentalen Gesundheit bedingen. Dabei könnten einerseits Armutsprozesse eine Rolle spielen, welche die Handlungsfreiheit einschränken [43]. Auch der Verlust wichtiger Arbeitsplatzfunktionen wie der Tagesstrukturierung, der identitätsstiftenden und sinngebenden Rolle des Arbeitsplatzes sowie der sozialen Teilhabe könnten einflussnehmend sein [44]. Dagegen postuliert die *Selektionshypothese* (auch *Umgekehrte Kausationshypothese*) die gegenteilige Wirkrichtung: Beschäftigte mit psychischen Störungen würden häufiger vom Arbeitgeber gekündigt, beispielsweise wegen Langzeit-Arbeitsunfähigkeit oder wegen Leistungseinbußen im Rahmen von Präsentismus. Sie hätten darüber hinaus aufgrund der gesundheitlichen Einschränkungen schlechtere Chancen, wieder in Arbeit zu gelangen [45], und die möglicherweise geringeren motivationalen Ressourcen für die Arbeitsplatzsuche könnten diese Wahrscheinlichkeit zusätzlich verringern [35]. Die Kausationshypothese findet u. a. Unterstützung in der groß angelegten Metaanalyse von Paul und Moser [35], die auch 87 Längsschnittstudien einbezieht: Hier zeigte sich, dass sich die psychische Gesundheit bei einem Arbeitsplatzverlust verschlechtert, während die Wiederaufnahme der Arbeit mit einer Verbesserung des psychischen Befindens einhergeht. Dies spricht dafür, dass die Arbeitslosigkeit die Belastungen tatsächlich kausal bedingt.

Obgleich Selektionsprozesse bisher weniger untersucht wurden, zeigten sich dennoch ebenfalls Hinweise darauf, dass Personen mit psychischen Belastungen häufiger arbeitslos werden, weniger wahrscheinlich einen neuen Arbeitsplatz aufnehmen und nach der Schullaufbahn seltener der Einstieg in den Arbeitsmarkt gelingt [35]. Hier scheinen Arbeitsmarktprozesse eine Rolle zu spielen, die Menschen mit psychischen Erkrankungen strukturell benachteiligen [46], was die Annahme der gegenteiligen Wirkrichtung von schlechterer psychischer Gesundheit auf die Entstehung und Aufrechterhaltung von Arbeitslosigkeit unterstützt. Kausations- und Selektionseffekte schließen sich jedoch nicht gegenseitig aus. Vielmehr können sie ineinanderwirken [47]: Ein Arbeitsplatzverlust bei psychisch erkrankten Menschen führt potenziell zur Verringerung von sozialen und leistungsbezogenen Verstärkern (siehe Verstärker-Verlust-Modell zur Entstehung und Aufrechterhaltung von Depression; [48]), wodurch sich die psychische Gesundheit weiter verschlechtern kann. Dadurch werden wiederum die Suche eines neuen Arbeitsplatzes und das erfolgreiche Antreten der Stelle erschwert.

Die Theorie der Kompensationseffekte besagt darüber hinaus, dass die Wahrscheinlichkeit, arbeitslos zu werden, je nach Bildungsgrad unterschiedlich verteilt ist [49]. So sind Menschen mit geringer schulischer bzw. beruflicher Qualifikation häufiger von Arbeitslosigkeit betroffen, was sie somit auch vulnerabler für die Entwicklung psychischer Störungen machen könnte.

## Behandlungsspezifische Aspekte

Die individuellen Folgen eines längeren Fernbleibens vom Arbeitsplatz aufgrund psychischer Störungen verdeutlichen neben den volkswirtschaftlichen Konsequenzen die Notwendigkeit entsprechender Behandlungsprogramme. Studien aus den letzten Jahren konnten zeigen, dass Psychotherapie hier sowohl effektiv als auch effizient ist. Es wird jedoch davon ausgegangen, dass weniger als 25 % der behandlungswilligen Personen mit einer psychischen Störung heute überhaupt eine Behandlung erhalten; bei affektiven und Angststörungen sind es jährlich nur ca. 6–9 % [24], obwohl Psychotherapie in den Behandlungsleitlinien der medizinischen Fachgesellschaften als alleinständige Maßnahme und/oder in Kombination mit Psychopharmakotherapie empfohlen wird. Diese beginnt außerdem meist erst mehrere Jahre nach Inzidenz und entspricht häufig nicht den Minimalanforderungen an eine adäquate Therapie [25]. Die Kosten-Nutzen-Analyse von Wunsch et al. ([17]; Datenquellen siehe Tab. 1) für die häufigen Angst- und affektiven Störungen zeigte, dass bei geschätzten Remissionsraten von 78 % (Angststörungen; [50]) bzw. 59 % (affektive Störungen; [51]) durch Therapie und einer geschätzten Behandlungswilligkeit von konservativ 33 % bzw. progressiv 50 % 65- bis 100-mal so viele Patient:innen erfolgreich behandelt werden könnten, als es derzeit der Fall ist. Geht man davon aus, dass die AU-Tage dadurch reduziert werden oder wegfallen, könnte insbesondere eine deutliche Reduktion der Bruttowertschöpfungskosten von 1,0–1,6 Mrd. € pro Jahr erreicht werden.^6^ Auch die Produktionsausfallkosten und die Kosten durch Krankengeldzahlungen könnten erheblich reduziert werden.

Insgesamt positive Kosten-Nutzen-Verhältnisse zeigten sich bei einer Behandlungsdauer von nur 10 Sitzungen bereits bei sehr geringer Behandlungswilligkeit und Remissionsrate, bei 25 Sitzungen ebenfalls noch bei konservativ geschätzten Werten. Auch eine aktuelle Kohortenstudie unter Verwendung von Daten der Betriebsrankenkassen konnte zeigen, dass tiefenpsychologisch fundierte und kognitiv-behaviorale Therapien die AU-Tage bei häufigen psychischen Störungen gleichermaßen um ungefähr 14 Tage im Vergleich zu vor der Behandlung reduzieren können [53]. Dies ist ein Hinweis auf Effektivität und Effizienz von Psychotherapie bei diesen Störungsbildern, die Autor:innen der Studie weisen jedoch selbst darauf hin, dass naturalistische, möglichst längsschnittliche Erhebungen notwendig sind, um die Schätzungen bestätigen zu können. Für die Major Depression konnte verschiedentlich auch in kontrollierten Studien eine Reduktion der AU-Tage durch Psychotherapie festgestellt werden (z. B. [54]). Gleiches gilt insbesondere für expositionsbasierte Ansätze bei Angststörungen, wobei eine aktuelle Multicenter-Studie bei Patient:innen mit Panik-, sozialer Angststörung, Agoraphobie oder multiplen spezifischen Phobien eine Überlegenheit von intensivierter expositionsbasierter Behandlung gegenüber der entsprechenden nichtintensivierten Behandlung hinsichtlich der Reduktion der AU-Tage feststellen konnte [55].

In Bezug auf weniger häufige, aber schwer zu behandelnde psychische Störungen wie Essstörungen zeigte sich in der Kosten-Nutzen-Analyse von Bode et al. [19] ebenfalls die Möglichkeit einer deutlichen Reduktion der indirekten Kosten durch kognitiv-behaviorale sowie tiefenpsychologisch fundierte Psychotherapie. Dies gilt neben den Kosten für Arbeitsunfähigkeit insbesondere für die krankengeldbezogenen Ausgaben, zumal die Krankengeldtage für AN und BN relativ zu anderen Störungsbildern besonders hoch sind (Tab. 1). Insgesamt bewegte sich das Kosten-Nutzen-Verhältnis unabhängig von der Behandlungswilligkeit für diese Störungsbilder zwischen 2,33 und 4,05, sodass die flächendeckende Implementation von evidenzbasierten Behandlungsprogrammen für diese Störungen auch wirtschaftlich sinnvoll erscheint. Gleiches gilt für die schwer zu behandelnde BPS, bei der das Kosten-Nutzen-Verhältnis beim Einsatz von dialektisch-behavioraler Psychotherapie mit 1,52 beziffert wird [20].

Naturalistische Studien lassen darüber hinaus vermuten, dass die Arbeitsfähigkeit durch Interventionen, die den Arbeitsplatz fokussieren, eher wiederhergestellt werden könnte [56]. So reduzierte eine arbeitsplatzbezogene kognitive Verhaltenstherapie im Rahmen eines interdisziplinären Versorgungsprojekts einer Psychotherapieambulanz und ausgewählter Krankenversicherungen die Rate der AU-Tage bei Erwerbstätigen mit depressiver Episode stärker als die herkömmliche Verhaltenstherapie. Unterdessen konnten die Depressivität und Symptomschwere in beiden Bedingungen gleichermaßen gesenkt sowie die Lebensqualität verbessert werden [54]. Eine groß angelegte niederländische Studie, die neben affektiven Störungen auch somatoforme Störungen, Angst- und Anpassungsstörungen einbezog, zeigte außerdem, dass Proband:innen, die eine arbeitsbezogene kognitive Verhaltenstherapie erhielten, mehr als 2 Monate früher vollständig an den Arbeitsplatz zurückkehrten, als es bei einer regulären kognitiven Verhaltenstherapie der Fall war [57]. Auch hier wurde in beiden Bedingungen eine vergleichbare Symptomreduktion erreicht.

Im Hinblick auf spezifisch arbeitsphobische Ängste konnte in einer kontrollierten Studie in der stationären Rehabilitation darüber hinaus gezeigt werden, dass eine kognitiv-behaviorale Gruppenintervention mit Fokus auf arbeitsbezogenes Problemlöse- und Stressmanagementtraining verglichen mit einer ablenkungsorientierten Freizeitgruppe zu einer stärkeren Verkürzung der Arbeitsunfähigkeitsdauer sowie einer optimistischeren Selbsteinschätzung der arbeitsbezogenen Bewältigungsfertigkeiten führte [58, 59].

Im Rahmen der genannten Interventionen werden über das störungsspezifische Vorgehen hinaus Störungsmodelle mit den Patient:innen erarbeitet, bei denen ein Fokus auf die psychosozialen Funktionen von Arbeit gelegt wird. Außerdem erfolgt die Planung von spezifischen Maßnahmen im Arbeitskontext und eine sukzessive Vorbereitung der Wiedereingliederung der Patient:innen. In diesem Rahmen werden auch mögliche Schwierigkeiten und ggf. Hilfeinstanzen identifiziert sowie gemeinsam entsprechende Bewältigungsstrategien, z. B. in Rollenspielen, herausgearbeitet [54]. Zentraler Aspekt ist insgesamt, dass der Arbeitsplatz soweit möglich als Kontext für bestimmte Behandlungsziele (z. B. Verhaltensaktivierung, Steigerung des Selbstwertgefühls) eingesetzt wird [57]. Bei Bedarf kann darüber hinaus Kontakt zu betrieblichen Instanzen wie Arbeitsmediziner:innen und Vorgesetzten aufgenommen werden und es können gemeinsame Gespräche mit den Behandler:innen angeregt werden, um die Wiedereingliederung zu erleichtern ([60]; im Detail). In weiteren, störungsspezifischen Therapiestudien sollte erprobt werden, inwiefern und unter welchen Anpassungen die Wirksamkeit dieser Maßnahmen auch auf andere als die genannten Störungsbilder übertragbar sein könnte.

## Fazit

Arbeitsunfähigkeit und ihre Folgen wie Erwerbsminderung stellen sowohl auf volkswirtschaftlicher Ebene als auch für die betroffenen Menschen eine erhebliche psychosoziale Belastung dar. Insbesondere häufige psychische Störungen wie affektive und Angststörungen sind dabei für volkswirtschaftliche Ausfälle in Milliardenhöhe verantwortlich, wobei bei selteneren psychischen Störungen wie Essstörungen und PTBS verhältnismäßig hohe indirekte Kosten aufgrund von Krankengeldzahlungen und Frühverrentungen entstehen. Auf individueller Ebene geht Erwerbslosigkeit ebenfalls mit erheblichen finanziellen Einschränkungen einher; etwa die Hälfte der Erwerbslosen sind nach aktuellen Erhebungen armutsgefährdet. Außerdem sind bei Erwerbsminderung und insbesondere bei Arbeitslosigkeit eine Verschlechterung der körperlichen und psychischen Gesundheit sowie erhöhte Mortalitäts- und Suizidraten zu beobachten. Als Ursachen hierfür werden Kausations- und Selektionseffekte diskutiert. Aktuelle Befunde zeigen, dass der Verlust des Arbeitsplatzes mit einer Verschlechterung der mentalen Gesundheit einhergeht, während sich diese bei Wiederaufnahme der Arbeit wieder verbessert, was dafür spricht, dass Arbeitslosigkeit diese Verschlechterung kausal bedingt. Auch Selektionseffekte werden allerdings beobachtet, d. h., Arbeitnehmende, die bereits psychische Belastungen aufweisen, verlieren mit höherer Wahrscheinlichkeit ihre Anstellung. Kausations- und Selektionseffekte können sich gegenseitig bedingen. Etablierte Therapieverfahren haben sich in gesundheitsökonomischen Studien als effektiv und effizient in der Behandlung häufiger psychischer Störungen wie Angst- und affektiver Störungen erwiesen, wobei arbeitsplatzbezogene Interventionen die Dauer der Arbeitsunfähigkeit nach aktuellen Studien stärker reduzieren könnten. In diesen Behandlungen werden arbeitsbezogene Modelle mit einbezogen und die Wiedereingliederung frühzeitig mit den Patient:innen geplant. Für die Generalisierbarkeit der Befunde bedarf es weiterer Forschung zu arbeitsplatzbezogenen Interventionen in naturalistischen Kontexten und bei weniger häufigen psychischen Störungen.

## Footnotes

1 Im Unterschied zur Erwerbsminderung bzw. -unfähigkeit, die sich auf das grundsätzliche Unvermögen bezieht, mindestens 6 h täglich erwerbstätig zu sein, adressiert die Arbeitsunfähigkeit nur die zuletzt ausgeübte Tätigkeit. Diese kann nicht mehr oder nur unter Gefahr der Verschlimmerung der Erkrankung ausgeübt werden. Arbeitslos ist dagegen, wer nicht in einem Beschäftigungsverhältnis steht sowie Eigenbemühungen unternimmt, die Beschäftigungslosigkeit zu beenden bzw. Vermittlungsbemühungen zur Verfügung steht.

2 In die AU-Statistiken der Krankenkassen fließen Kurzzeit-Arbeitsunfähigkeiten von bis zu 3 Tagen, die nicht durch Krankschreibungen von Ärzt:innen erfasst werden, nicht mit ein.

3 In den Angaben der Krankenkassen fehlen Dispersionsmaße, die eine detailliertere Beschreibung der Entwicklung ermöglichen würden.

4 Als indirekte Kosten bezeichnet man die sekundären Folgen psychischer Störungen, welche aus dem Ressourcenverlust aufgrund dieser resultieren (z. B. aufgrund von Arbeitsunfähigkeit; [15]), während direkte Kosten unmittelbare Ausgaben im Gesundheitssystem aufgrund der Störung bezeichnen (z. B. für Medikamente oder ambulante/stationäre Behandlungen).

5 Als armutsgefährdet gelten Personen, deren Einkommen weniger als 60 % des mittleren Einkommens beträgt.

6 Es wurde von Komplianzraten von 85 % für Angststörungen und 80 % für affektive Störungen ausgegangen [52].
